# The potential of foliar application of nano-chitosan-encapsulated nano-silicon donor in amelioration the adverse effect of salinity in the wheat plant

**DOI:** 10.1186/s12870-022-03531-x

**Published:** 2022-03-26

**Authors:** Shokoofeh Hajihashemi, Shadi Kazemi

**Affiliations:** Plant Biology Department, Faculty of Science, Behbahan Khatam Alanbia University of Technology, 63616-63973 Khuzestan, Iran

**Keywords:** Antioxidant mechanism, Chitosan, Nano-fertilizer, Osmotic adjustment, Salinity stress, Silicon

## Abstract

**Background:**

Nano-materials ameliorate the adverse effect of salinity stress on the physiological and biochemical processes in plants. The present investigation was designed to evaluate the physiological mechanisms through which a nano-chitosan-encapsulated nano-silicon fertilizer (NC-NS) can ameliorate the adverse effect of salinity stress on the wheat plants, and compare it with nano-chitosan (NC) and nano-silicon (NS) application. Nano-silicon was encapsulated with a chitosan-tripolyphosphate (TPP) nano-matrix by ionic gelation method for its slow release. The wheat plants were exposed to foliar application of distilled water, NC, NS, and NC-NS with two NaCl irrigation levels at 0 (distilled water) and 100 mM.

**Results:**

The foliar application of NC, NS, and NC-NS induced a significant increase in the function of enzymatic and non-enzymatic antioxidant systems of the wheat plants to equilibrate cellular redox homeostasis by balancing H_2_O_2_ content in the leaves and roots, as compared with salt-stressed plants without treatment. The plant's foliar-sprayed with NC, NS, and NC-NS solution exhibited a significant increase in the molecules with osmotic adjustment potentials such as proline, free amino acids, glycine betaine, and sugars to protect cells against osmotic stress-induced by salinity. The observed increase in the antioxidant power and osmoregulatory at NC, NS, and NC-NS application was accompanied by the protection of lipid membrane, proteins and photosynthetic apparatus against salinity stress.

**Conclusion:**

In the present study, the beneficial role of NC, NS, and NC-NS application, particularly NC-NS, in alleviating the adverse effect of salinity stress on antioxidant systems and osmotic adjustment in wheat is well documented. An overview of the result of present study assists researchers in providing a potential solution for this increasing salinization threat in crops.

**Graphical abstract:**

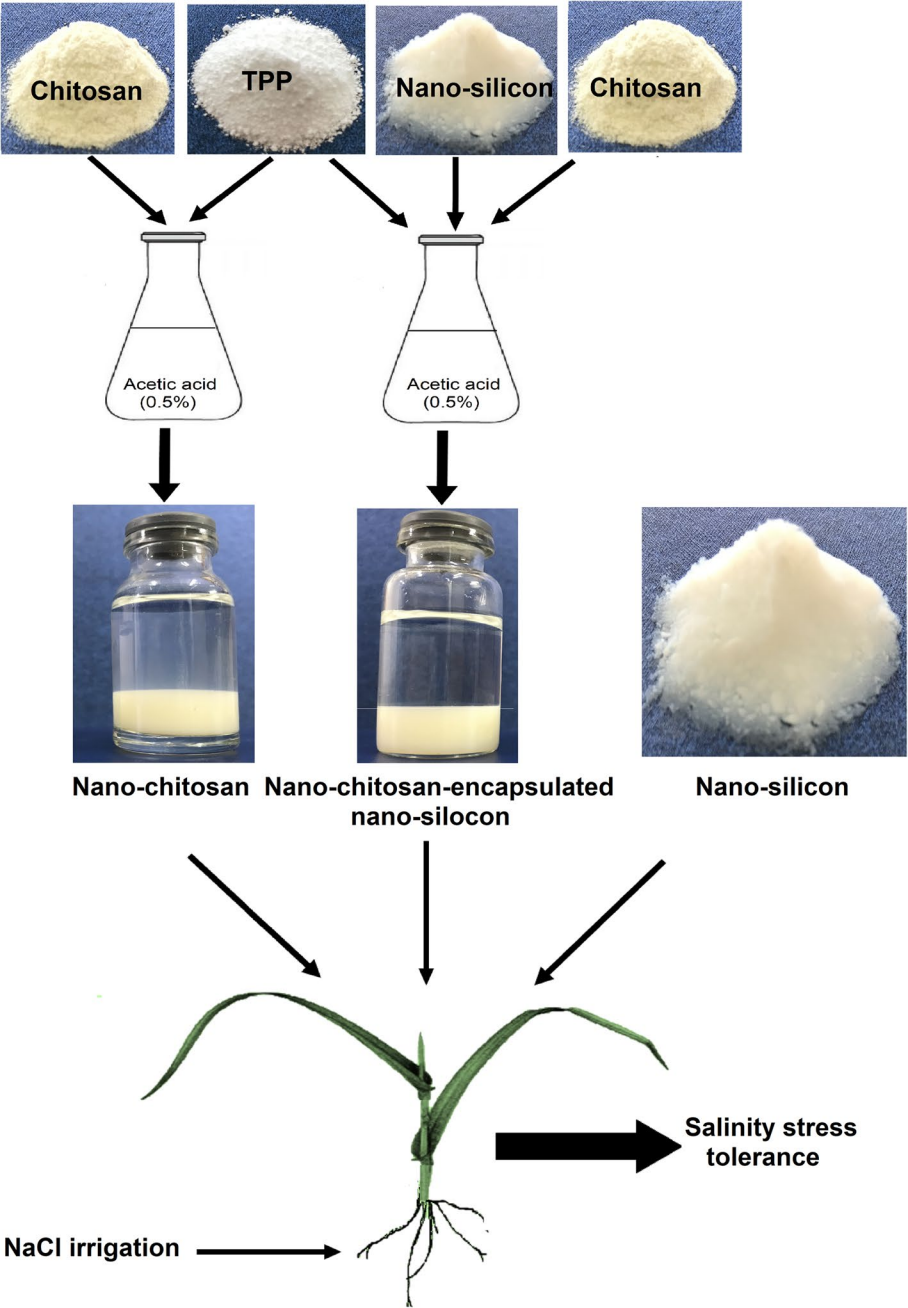

## Background

Salinity stress poses a threat to the world’s food security due to adversely affecting both crop yield and quality, which has been introduced as one of the most important factors contributing to crop losses worldwide [1, 2]. Considering the increasing threat of salinity stress, it is need time, to find avenues for developing salinity tolerance in crops. To alleviate the adverse effect of soil salinization on crop yield and promote sustainable agriculture, the utilization of foliar fertilizer nutrients is an acknowledged method. At present, a variety of research has been performed on foliar fertilization to moderate the adverse effect of soil salinization and improve salt tolerance in crops [2–4]. Supplementary foliar fertilization can deliver nutrients directly to the target organs through aerial parts of the plant, thereby helping to mitigate the negative impacts of stress [2]. In recent years, silicon (Si) fertilization is gaining increased attention to alleviate the adverse effect of salinity stress in plants [3, 4]. Si evolved altered mechanisms such as photosynthesis, antioxidant systems, osmolytes accumulation, and nutrient balance to counter salinity stress in higher plants such as wheat (*Triticum aestivum* L.) and rice (*Oryza sativa* L.) [5–8].

Wheat (*Triticum aestivum* L.) is the main cereal crop and is a staple food of the major population, which is widely grown worldwide [9–11]. In a number of *Poaceae* species, sustained availability of plant to Si is vital for precisely maintaining the plant growth [12]. One beneficial effect of Si under salinity stress has been reported concentrating sodium ions in the apoplast and enhancing photosynthetic pigments and leaf area in salt-treated plants [6, 13]. Repression of Na^+^ transport in wheat plants growing in salinized solutions supplied with Si improves salt tolerance [6, 12]. Nano-fertilizers may be more effective than regular fertilizers in enhancing plant nutrition and protecting them from environmental stress [14–16]. The regular Si fertilizers are generally low bioavailable, while nano-Si fertilizer with smaller particle size can easily penetrate the leaf cells and function more directly [17]. Nano-Si exhibited a great potential in reducing the adverse effect of UVB-stress in wheat (*Triticum aestivum* L.) seedlings and worked better than bulk Si, which can be related to its greater availability [18].

In recent years, the application of biomaterials such as biopolymer chitosan with multiple advantages of being safe, inexpensive, and easy producted and application has been enhanced worldwide. The chemical structure of chitosan easily allows the introduction of special molecules to design polymers for specific applications [19]. In particular, an increasing number of researchers has been investigating the effects of nano-chitosan-based fertilizers on plants under regular and stressful condition [20, 21]. A developed chitosan-Si nano-fertilizer exhibited a slow release of Si and promoted growth and yield in maize crops. The foliar application of chitosan-Si nano-fertilizer promoted plant antioxidant-defense and photosynthesis efficiency in maize [22]. NS effectively improved the growth and photosynthesis, and reduced the oxidative stress in wheat (*Triticum aestivum* L.) plants under cadmium stress [23]. Besides, the application of  chitosan plus Si increased the growth and plant water status, along with the physiological trials and yield attributes in the wheat plants under drought stress [24].

Up to our knowledge, an investigation has been performed on the efficiency of chitosan nano-matrix encapsulated bulk Si in maize under regular conditions [22], while there is no similar reports on wheat plants. Accordingly, the present study was designed to study the effect of nano-chitosan-encapsulated nano-Si fertilizer on the wheat plants under both regular and stress conditions. For this purpose, physiological and biochemical aspects of the plant response to a nano-chitosan-encapsulated nano-Si donor have been evaluated under regular and salinity stress conditions. Therefore, our aims were to: (1) investigate whether nano-Si (NS) or nano-chitosan (NC) or nano-chitosan-encapsulated nano-Si (NC-NS) application are more effective in alleviating salinity stress in wheat, (2) investigate comparative impact of NS or NC or NC-NS application on antioxidants systems (3) elucidate mechanisms through which NS or NC or NC-NS application alleviate salinity stress in wheat.

## Results

**Fig. 1:**
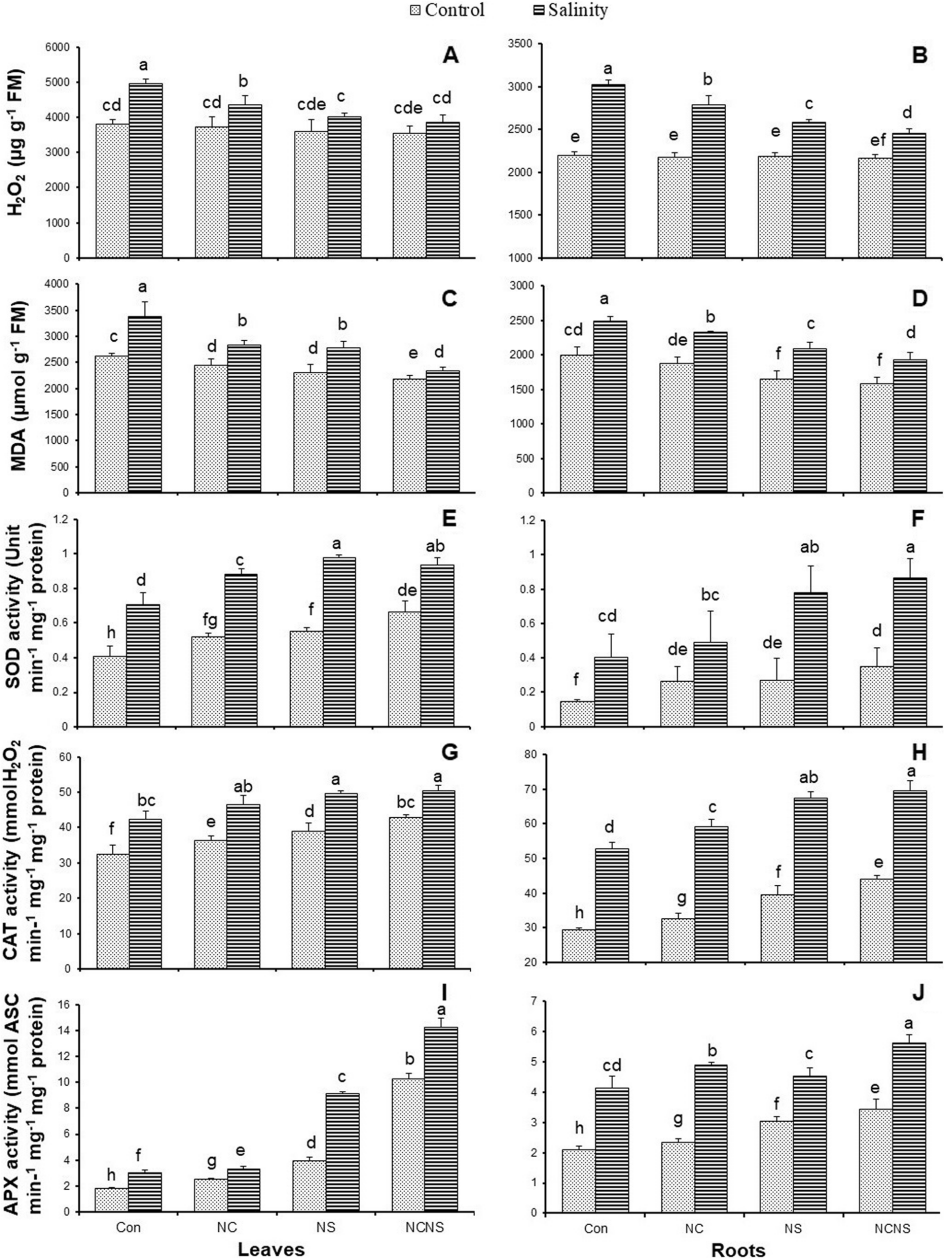
(**A** and **B**) H_2_O_2_, (**C** and **D**) malondialdehyde (MDA), (**E** and **F**) superoxide dismutase (SOD) activity, (**G** and **H**) catalase (CAT) activity, and (**I** and **J**) ascorbate peroxidase (APX) activity in the leaves and roots, respectively, of the wheat plants at nano-chitosan (NC), nano-silicon (NS) and nano-chitosan-encapsulated nano-silicon (NC-NS) application subjected to salinity stress at 0 (distilled water) and 100 mM NaCl. Columns with the same lower-case letters do not differ significantly at *p* ˂ 0.05

Salinity stress significantly increased the H_2_O_2_ accumulation in the leaves and roots, while the NC, NS, and NC-NS treatment had no significant effect on the H_2_O_2_ value, as compared with control (Fig. 1A and B). The NC, NS, and NC-NS application suppressed the H_2_O_2_ accumulation in the leaves and roots in the salt-stressed plants. Application of NC-NS was the most effective treatment in reducing the H_2_O_2_ accumulation in the leaves and roots of salt-stressed plants (Fig. 1A and B). In parallel with the observed H_2_O_2_ accumulation, the lipid peroxidation increased in the salt-stressed plants, as represented by an increase in the MDA value in the leaves and roots (Fig. 1C and D). The salt-stressed plants given NC, NS, and NC-NS treatment showed lower lipid peroxidation levels than plants without treatment (Fig. 1C and D). The SOD activity of salt-stressed and NC, NS, and NC-NS-treated plants exhibited the same trends. Salinity stress significantly improved the SOD activity of leaves and roots in parallel with the H_2_O_2_ level (Fig. 1E and F). The application of NC, NS, and NC-NS significantly increased the activity of SOD in the presence or bsence of NaCl in both leave and roots, with the greatest increase achieved at both NS, and NC-NS application (Fig. 1E and F). Both salinity stress and NC, NS, and NC-NS application significantly improved the activity of CAT and APX enzymes in the leaves and roots, with their greatest activity observed at NC-NS application in presence of NaCl (Fig 1G-J).

**Fig. 2:**
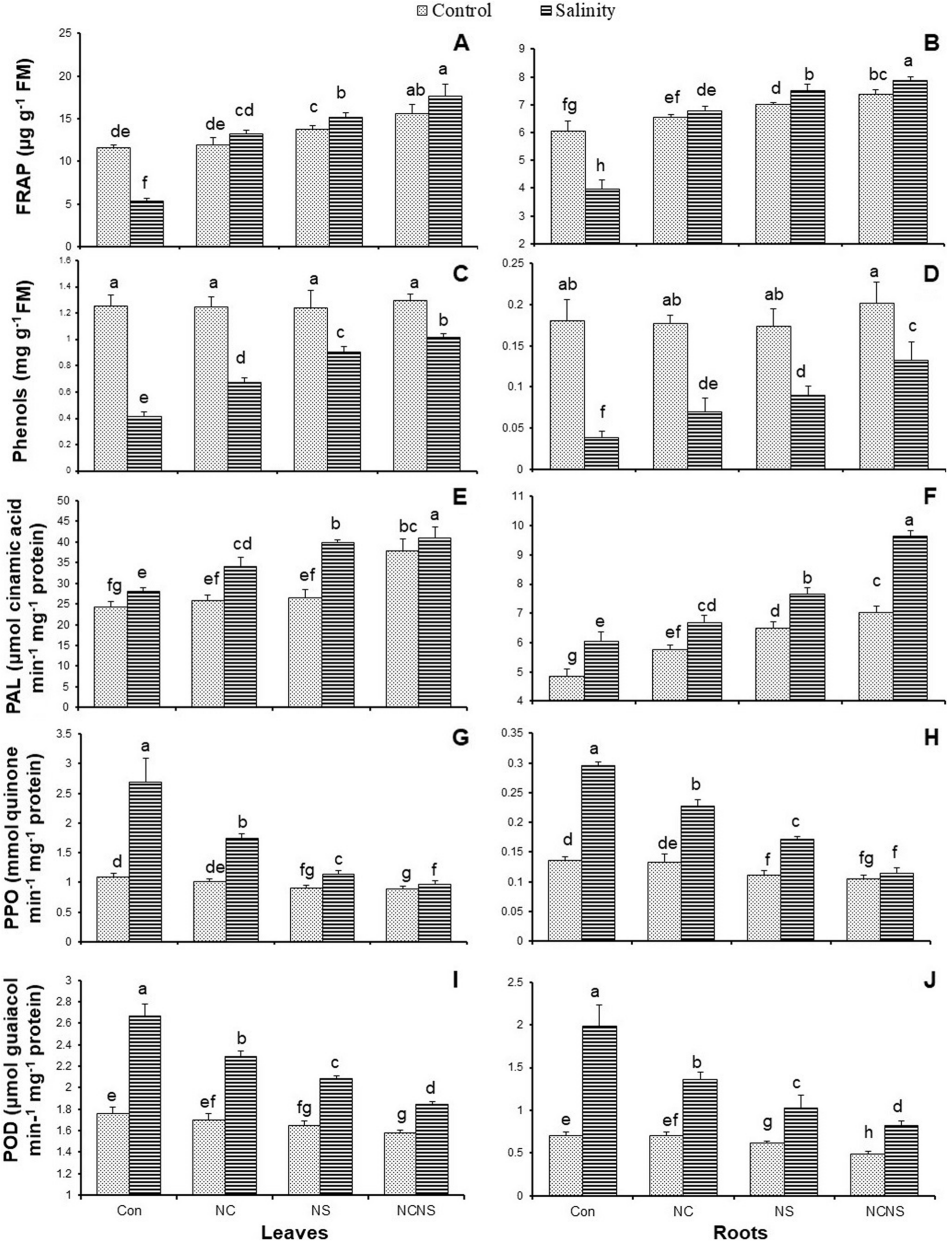
(**A** and **B**) Total antioxidant power (FRAP), (**C** and **D**) phenols, (**E** and **F**) phenylalanine lyase (PAL) activity, (**G** and **H**) polyphenol oxidase (PPO) activity, and (**I** and **J**) guaiacol peroxidase (POD) activity in the leaves and roots, respectively, of the wheat plants at nano-chitosan (NC), nano-silicon (NS) and nano-chitosan-encapsulated nano-silicon (NC-NS) application subjected to salinity stress at 0 (distilled water) and 100 mM NaCl. Columns with the same lower-case letters do not differ significantly at *p* ˂ 0.05

The total antioxidant activity, represented as FRAP, was significantly reduced in the leaves and roots by NaCl irrigation (Fig. 2A and B). In opposite, the application of NC, NS, and NC-NS increased the FRAP value in the leaves and roots of both stressed and non-stressed plants (Fig. 2A and B). Under irrigation with NaCl, the FRAP value had the greatest value at NC-NS application (Fig. 2A and B). The phenols values in the leaves and roots in the plants treated with NC, NS, and NC-NS showed no significant changes, while salinity stress significantly decreased phenols accumulation in the leaves and roots (Fig. 2C and D). The application of NC, NS, and NC-NS significantly increased the content of phenols in the leaves and roots to a level higher than at the same NaCl concentration without treatment, with the greatest increase achieved at the NC-NS application (Fig. 2C and D). In opposite with the reduction of phenolic compounds value in the NaCl-irrigated plants, the PAL activity significantly increased in response to salinity stress in the leaves and roots (Fig. 2E and F). The activity of PAL showed a significant increase in the leaves at NC-NS application, and in the roots at the NC, NS, and NC-NS application. At the NC, NS, and NC-NS application, the activity of PAL in the leaves and roots of NaCl-irrigated plants were greater than the NaCl irrigation without treatment (Fig. 2E and F). Under salinity stress, the activity of PPO and POD showed a significant increase in the leaves and roots, in the opposite trend with the phenols content (Fig. 2G-J). The activity of PPO and POD showed a slight but significant reduction in the leaves and roots at NS, and NC-NS application. In contrast to salinity stress, the NC, NS, and NC-NS treatment in presence of NaCl reduced the activity of PPO and POD in the leaves and roots less than their values in the salinity stress without treatment (Fig. 2G-J). The greatest reductions in the PPO and POD activity were observed at NC-NS application relative to NaCl irrigation without treatment (Fig. 2G-J).

**Fig. 3:**
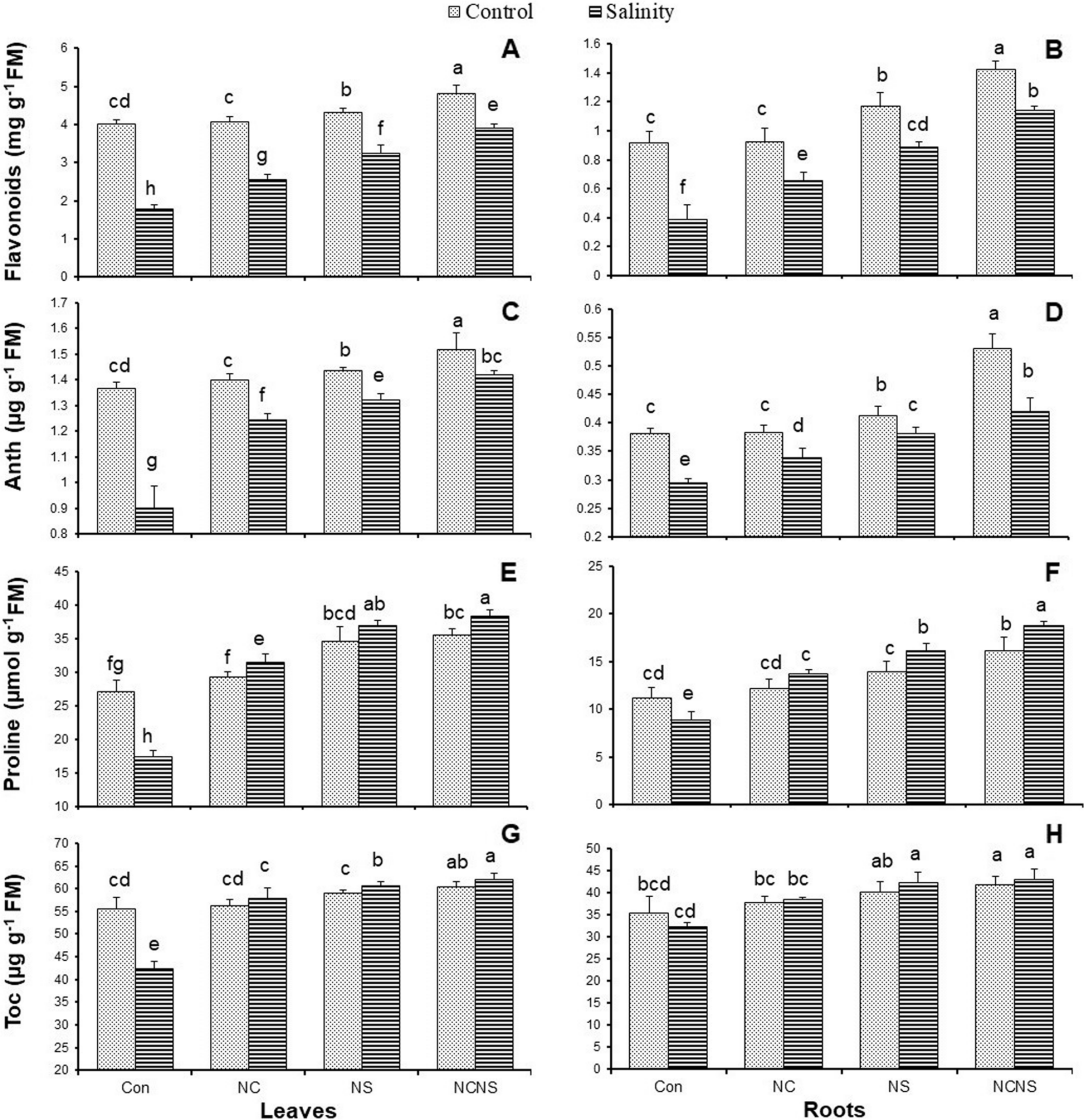
(**A** and **B**) Flavonoids, (**C** and **D**) anthocyanins (Anth), (**E** and **F**) proline, and (**G** and **H**) α-tocopherol (Toc) in the leaves and roots, respectively, of the wheat plants at nano-chitosan (NC), nano-silicon (NS) and nano-chitosan-encapsulated nano-silicon (NC-NS) application subjected to salinity stress at 0 (distilled water) and 100 mM NaCl. Columns with the same lower-case letters do not differ significantly at *p* ˂ 0.05

Both flavonoids and anthocyanins in the leaves and roots at the NS, and NC-NS application significantly increased greater than their values at the NC application and control plants (Fig. 3A-D). Salinity significantly reduced the amounts of flavonoids and anthocyanins in both leaves and roots, less than control. The application of NC, NS, and NC-NS significantly increased the content of flavonoids and anthocyanins in the leaves and roots to a level higher than at the same NaCl concentration without treatment, with the greatest increase achieved at the NC-NS application (Fig. 3A-D). Similar to flavonoids and anthocyanins, the proline contents of leaves and roots significantly decreased in the salt-stressed plants (Fig. 3E and F). Opposite to salinity stress, the NC, NS, and NC-NS application improved the proline value of both leaves and roots in the presence and absence of NaCl (Fig. 3E and F). The application of NC-NS in the salt-stressed plants was most effective in increasing the accumulation of proline in the leaves and roots (Fig. 3E and F). In the same as proline, salinity stress resulted in a significant decrease in α-tocopherol content, while the application of NC, NS, and NC-NS with or without NaCl significantly increased that to a level higher than its value in the salt-stressed plants (Fig. 3G and H). Exogenous NC-NS promoted the greatest accumulation of α-tocopherol in the leaves and roots and reduced the adverse effect of salinity on that (Fig. 3G and H).

**Fig. 4:**
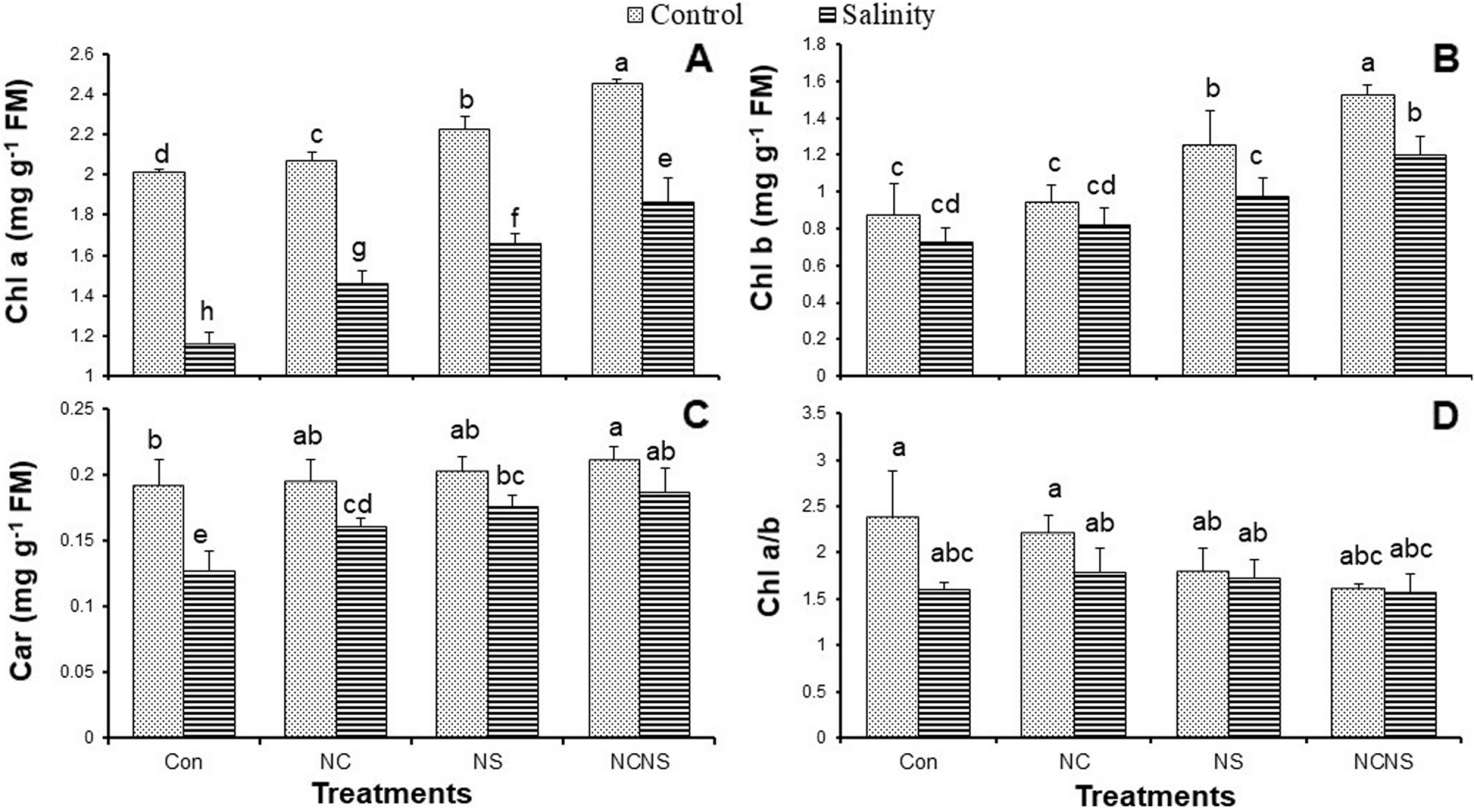
(**A**) Chlorophyll a (Chl a), (**B**) Chl b, (**C**) carotenoids (Car), and (**D**), and Chl a/b ratio in the wheat plants at nano-chitosan (NC), nano-silicon (NS) and nano-chitosan-encapsulated nano-silicon (NC-NS) application subjected to salinity stress at 0 (distilled water) and 100 mM NaCl. Columns with the same lower-case letters do not differ significantly at *p* ˂ 0.05

Measurements of photosynthetic pigments showed that the values of Chl a and b and carotenoids were significantly decreased in the salt-stressed plants, relative to control (Fig. 4A-C). The NC, NS, and NC-NS application, in the absence of NaCl, promoted an increase in Chls, while no significant changes were achieved in the carotenoid content. The application of NC, NS, and NC-NS to the salt-stressed plants significantly increased the value of photosynthetic pigments greater than their values at NaCl alone, with the highest increase observed at NC-NS with NaCl (Fig. 4A-C). The ratio of Chl a/b decreased in response to the NC, NS, and NC-NS application with or without NaCl, with the greatest reduction observed at NC-NS with NaCl (Fig. 4D).

**Fig. 5:**
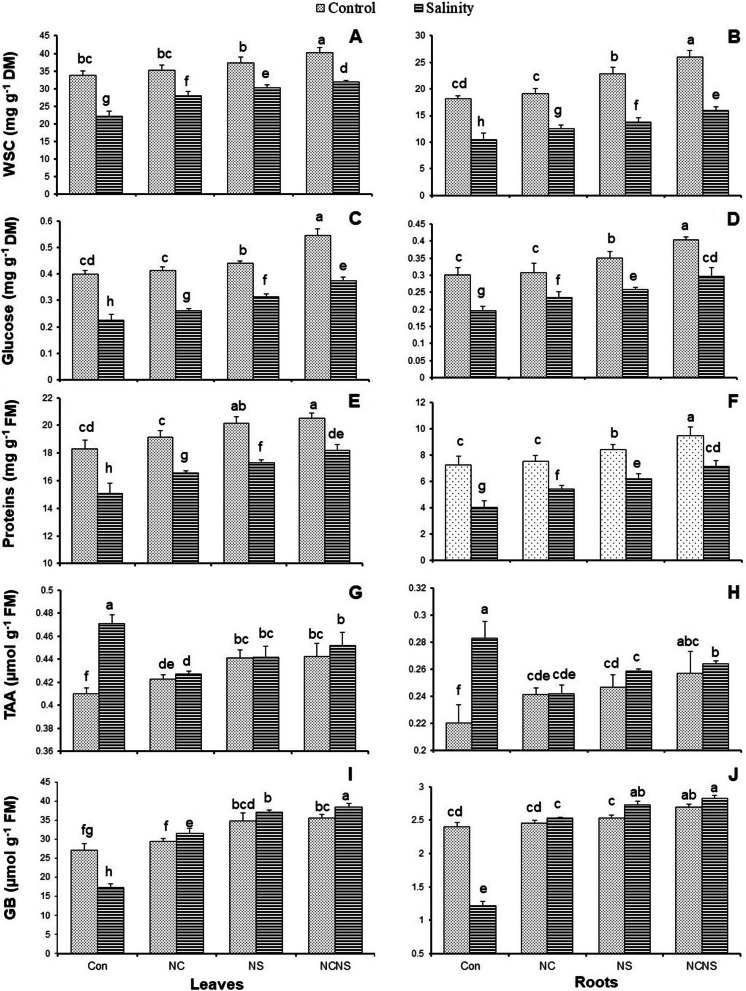
(**A** and **B**) Water-soluble carbohydrates (WSC), (**C** and **D**) glucose, (**E** and **F**) protein, (**G** and **H**) free amino acids (FAA), and (**I** and **J**) glycine betaine (GB) in the leaves and roots, respectively, of the wheat plants at nano-chitosan (NC), nano-silicon (NS) and nano-chitosan-encapsulated nano-silicon (NC-NS) application subjected to salinity stress at 0 (distilled water) and 100 mM NaCl. Columns with the same lower-case letters do not differ significantly at *p* ˂ 0.05

Along with the observed decrease in the photosynthetic pigments in the salt-stressed plants, the WSC and glucose contents in the leaves and roots also showed a significant reduction (Fig. 5A-D). The NC, NS, and NC-NS application promoted the WSC and glucose accumulation in the leaves and roots in the presence or absence of NaCl, as compared with the same NaCl treatment (Fig. 5A-D). The WSC and glucose contents in the leaves and roots showed the greatest values at the NC-NS application with or without NaCl (Fig. 5A-D). Salinity stress adversely affected the proteins of the wheat plants as shown by the reduction in the proteins of leaves and roots (Fig. 5E and F). The NC, NS, and NC-NS treatment increased the proteins value in the leaves and roots, compared to control plants. In the presence of salinity stress, the NC, NS, and NC-NS treatment significantly improved the proteins accumulation in the leaves and roots, compared with the NaCl irrigation without treatment (Fig. 5E and F). The application of NC-NS was the most effective in reducing the adverse effects of salinity on the proteins value (Fig. 5E and F). Salinity stress promoted a significant increase in the value of free amino acids of leaves and roots (Fig. 5G and H). Similar to salinity stress, the application of NC, NS, and NC-NS with or without NaCl significantly increased the value of free amino acids of leaves and roots, while the greatest increase was observed at the NaCl irrigation without treatment (Fig. 5G and H). Similar to proline, salinity stress induce a significant reduction in the glycine betaine accumulation in the leaves and roots, while the NC, NS, and NC-NS application in the presence or absence of NaCl improved it in the leaves and roots, with the greatest increase was obtained at the NC-NS application (Fig. 5I and J).

## Discussion

The application of environmentally friendly and stress-alleviating macro-elements is a technique to ameliorate the adverse effects of stress on plants. The beneficial role of Si, as the second most abundant element in the earth’s crust, in alleviating salinity stress in the wheat plants is well known [5, 7, 8]. However, the alleviating mechanisms of bulk Si alone or encapsulated with nano-chitosan matrix were studied in several crops under regular or salinity conditions [3, 5, 6, 13, 21, 22, 25, 26], the potential of nano-Si encapsulated with nano-chitosan matrix, and associated mechanism in alleviating the adverse effect of salinity stress has not been earlier studied on plant species, particularly wheat. Based on a report on the NC-NS potential in controlling the oxidation process of stored button mushroom at 4˚C [27], the present study was designed to evaluate how the antioxidant system of salt-stressed wheat plants responds to NC-NS application, and consequence physiological, and biochemical processes were evaluated to better understand the associated mechanisms.

The results of present study were in accordance with the hypothesis that salinity stress leads to oxidative stress due to the high accumulation of reactive oxygen species (ROS), which adversely suppressed the regular physiological and biochemical processes of wheat plants [1, 26, 28]. H_2_O_2_ is one of the most stable and major ROS [6, 29], which showed a significant increase in the NaCl-irrigated wheat plants. In opposite, the NC, NS, and NC-NS applications reduced the H_2_O_2_ value in the NaCl-irrigated plants, with respect to the NaCl irrigation without treatment. Attribution has been reported between the high accumulation of H_2_O_2_ in the salt-stressed plants and lipid peroxidation represented by a high accumulation of MDA [6], which was in accordance with the results of present study. Salinity stress increased the end-product of lipid peroxidation, i.e., MDA content, in the leaves and roots of the wheat plant, which was alleviated by the NC, NS, and NC-NS application. This appears to be supported by previous reports, based on the role of chitosan and Si in scavenging ROS and reducing the MDA accumulation in the salt-stressed wheat plants [26, 30]. The SOD, CAT, and APX enzymes showed a similar tendency in response to salinity stress in the wheat plants. Exogenous NC, NS, and NC-NS alleviated oxidative injury in the wheat plants by increasing the activity of antioxidant enzymes such as SOD, CAT, and APX to directly scavenge H_2_O_2_, which is represented in the reduction of H_2_O_2_ accumulation and improving the integrity of cell membranes in the salt-stressed plants. There are reports based on the active role of Si and chitosan in maintaining antioxidants enzymes activity in the wheat plants under salt stress [26, 30]. Overall, the highest activity of SOD, CAT, and APX enzymes and lowest H_2_O_2_ and MDA contents were observed in the NC-NS application.

The findings of present study confirmed the higher antioxidant activity in the NC, NS, and NC-NS-treated wheat plants, in the presence or absence of NaCl, represented in the increased-FRAP level in the leaves and roots. In opposite, salinity stress decreased the FRAP value in the leaves and roots of the wheat plants. It is well documented that the improvement of enzymatic and non-enzymatic antioxidant systems in the salt-stressed plants suppress oxidative stress due to scavenging free radicals and inhibition of oxidative stress-induced injuries in cells [1, 6]. Wheat is a Si-accumulating species, and it has already reported that Si improved both enzymatic and non-enzymatic antioxidant systems in the salt-stressed wheat plants [26]. The plants contain several non-enzymatic antioxidants including phenolic compounds, flavonoids, anthocyanins, proline, tocopherols, and carotenoids [29, 31, 32]. Polyphenols, with an important role in ROS scavenging, could be also substrates for different peroxidase enzymes, involved in the defense system against stress conditions. Phenols can detoxify H_2_O_2_ by donating electrons to guaiacol-type peroxidases under stress conditions [27, 32, 33]. A decrease was noticed in the phenols value in the salt-stressed wheat plants, which was in the opposite trend to the PAL activity. On the other hand, the possible reason for the observed reduction in the value of the phenolic compounds might be explained by the observed increase in the POD and PPO, which involve detoxifying ROS by oxidation of phenolic compounds as substrate [27, 32]. In this context, it is imperative to mention that reduction of POD activity in button mushroom-treated with nano-Si-chitosan was accompanied by a less browning process and longer shelf-life, due to reduction of phenols oxidation [27]. The application of NC, NS, and NC-NS suppressed the adverse effect of salinity stress on the phenolic compounds, which was in parallel with the aroused PAL activity. The activity of PPO and POD in response to the NC, NS, and NC-NS application was greater than control plants but less than salt stress without treatment. PAL, POD, and PPO are key enzymes in lignin biosynthesis, which the application of salicylic acid nano-chitosan application increased their activity in maize plants [34]. Lignin, as a major hydrophobic component of the cell walls in plants, renders resistance to the cell walls and makes plant cell wall-less accessible to degrading enzymes [34]. Altogether, the application of NC, NS, and NC-NS might have also aroused the plant antioxidant defense against salinity stress through regulating phenols accumulation and the activity of associated enzymes of PAL, POD, and PPO in the leaves and roots of the wheat plants.

In accordance with the phenolic compounds, a decrease was achieved in the flavonoids and anthocyanins value in the salt-stressed wheat plant, which was reversed in the NC, NS, and NC-NS application. The possible reason for the observed increase in the flavonoids and anthocyanins value might be explained by the observed increase in the antioxidant power of NC, NS, and NC-NS-treated plants under stress conditions. The alleviation of oxidative stress in the stressed-plants triggered the accumulation of flavonoids and anthocyanins with ROS scavenging potential [29, 32, 35]. In parallel with phenols, flavonoids and anthocyanins, the NC, NS, and NC-NS application triggered the biosynthesis of other organic antioxidants including proline, amino acids and α-tocopherol. Plants exposure to environmental stress induces synthesize diverse metabolites including specific amino acids, such as proline with the major functions in antioxidant defense to scavenge free radicals, osmotic adjustment to maintain cell water stability and stabilize macromolecule, storage of carbon and nitrogen for use during stress regimes [9, 26, 32, 36]. Furthermore, proline with a function as a ROS scavenger reduces photodamage in the thylakoid membranes under stress conditions [36]. The excess level of proline due to the NC, NS, and NC-NS application in the salinity stressed-plants might partially explain the induced increase in the FRAP value and reduction of MDA accumulation, which confirms the previous reports based on the role of Si and chitosan in salinity amelioration in the wheat plants [26, 30]. α-Tocopherol, as the most active form of vitamin E, is capable to scavenge oxygen radicals and lipid peroxides, and quenching singlet oxygen [28]. In a similar trend to proline, α-tocopherol value increased in response to the NC, NS, and NC-NS application in the presence or absence of NaCl, which may be contributed to protecting the wheat plants from oxidative damages due to its antioxidant potential [32]. The result of present research revealed a reverse relation between the α-tocopherol value and the MDA accumulation, which confirms the previous findings based on the increased-α-tocopherol value under stress conditions reduced the adverse effect of oxidative damage on lipid membrane [32, 37]. In summary, the salinity stress-induced increase in ROS accumulation-accompanied with lipid membrane peroxidation was alleviated by NC, NS, and NC-NS application through stress-induced enzymatic and non-enzymatic antioxidant systems. The activation of antioxidant systems in the NC, NS, and NC-NS application in stressed plants was accompanied by observed lower H_2_O_2_ value and stabilizing lipid membrane. The most effective treatment in reducing the adverse effect of salinity stress in the leaves and roots of wheat plants was the NC-NS application, which might be the slow release of NS. Kumaraswamy et al. [22] has already acclaimed that the slow release of salicylic acid from salicylic acid-chitosan nanoparticles has significantly amended physiological and biochemical responses in maize for plant growth and yield, and diseases control compared to sole SA application.

Under salinity stress, the photosynthetic pigments were adversely affected, while the NC, NS, and NC-NS applications significantly amended them. The supplementation of Si improved the potassium content in the salt-stressed what plants [26], which can decrease photo-oxidative damage to chloroplasts and suppress chlorophylls degradation by maintaining a high pH level in the stroma [6]. Besides, the alleviation of oxidative stress in stressed-plant by Si application triggered the accumulation of chlorophylls and carotenoid contents in wheat [5, 38]. In parallel with strengthened antioxidant systems in the NC, NS, and NC-NS application under salinity stress conditions, the salinity stress-induced photo-damage to photosynthetic apparatus suppressed in response to the NC, NS, and NC-NS application. One of the most common indicators of oxidative stress-induced photo-damage in plant cells is a reduction of photosynthetic pigments resulting in impairment of carbohydrates biosynthesis [29]. In the same trend to photosynthetic pigments, the NC, NS, and NC-NS application increased glucose and WSC to a level greater than the same NaCl treatment. Abdel-Haliem et al [6]. has acclaimed that the nano-silicon application induced a significant increase in the total soluble carbohydrates in rice plants, which indicates their effective involvement in osmotic adjustment. Besides, the application of chitosan had beneficial effect on the chlorophylls and photosynthetic rate in wheat under salinity stress, followed by an increase in the carbohydrates [30]. The most effect treatment in reducing the adverse effect of salinity stress on photosynthetic pigments was determined as the NC-NS application.

Free radicals like H_2_O_2_-induced by salinity stress conjugate to proteins and result in their destruction [39, 40], which provided evidence of reduction of proteins value in the present study. In opposite, the impact of NC, NS, and NC-NS application on proteins under stress conditions might be due to both strengthened antioxidant systems and H_2_O_2_ scavenging leading to the proteins protection [35, 39, 41, 42]. Salinity stress involves both ionic stresses, resulting from high concentrations of salt ions within cells, and osmotic stress, by limiting water absorption. A variety of mechanisms evolves in plants to acclimatize to these unfavorable conditions. Sugars, Amino acids, polyols, and various quaternary ammonium compounds with low molecular weight known may accumulate in plant cells as compatible solutes to improve the ability to retain cell water without affecting the normal metabolism [43]. The reduction of protein contained in the salt-stressed wheat plants was accompanied by aroused free amino acids, which might provide an evidence of protein degradation and biosynthesis inhibition in response to stress. Di Martino et al [43]. reported a significant increase in the free amino acids and glycine betaine in the leaf of spinach to increase salinity stress tolerance. The free amino acid value in the salt-stressed wheat plants was significantly greater than that in control plants and the NC, NS, and NC-NS application in the presence or absence of NaCl, which is a partial feedback of protein degradation. The observed increase in the free amino acids in the NC, NS, and NC-NS application with or without NaCl can be referred to their antioxidant power to scavenge free radicals, and osmotic adjustment potential to maintain the cell osmotic pressure higher than the outer medium to induce water absorbance under stress condition [6, 29]. Proline and glycine betaine are the most known compatible solutes in plants for withstanding the prevailing drought stress [36]. Glycine betaine has been shown to protect the activities of enzymes such as rubisco and malate dehydrogenase, and cell membrane from the adverse effects of stress [36]. The application of NC, NS, and NC-NS reversed the effect of salinity stress on glycine betaine, which was followed by a significant increase in its value in the NaCl irrigation, which triggered the maintain of the lipid membrane and osmotic adjustment [36]. In general, the results exhibited that the beneficial effects of NC-NS treatment on reducing the adverse effect of salinity stress in wheat was higher than the sole NC or NS application.

## Conclusions

The present study confirmed that the application of NC, NS, and NC-NS significantly improved salt tolerance in the wheat plant, by improving the enzymatic and non-enzymatic antioxidant systems to limit the adverse effect of stress on oxidation of lipid membrane, photodamage in photosynthetic apparatus, and proteins macromolecule. Overall, a positive correlation was detected between the improvement of antioxidants and compatible solutes with the improvement of salinity tolerance in the wheat plant, with the greatest positive effect achieved at the NC-NS application. These findings suggest the potential advantages of further studies on the nano-Si encapsulated with nano-chitosan matrix to improve environmental stress in crop plants.

## Materials and methods

### Description of the nano-chitosan and nano-chitosan-nano-Si preparation

**Fig. 6:**
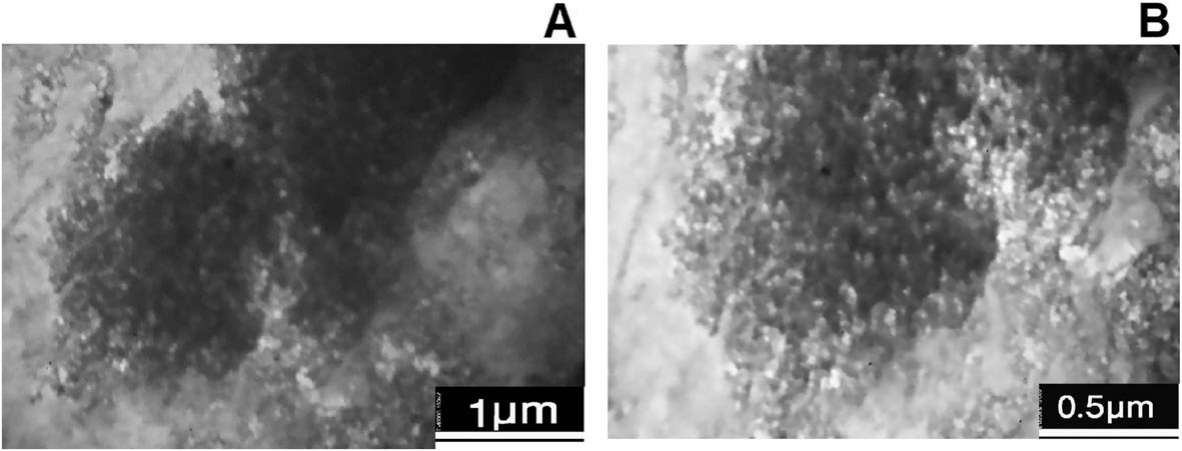
The TEM images of nano-chitosan-encapsulated nano-silicon (NC-NS) particles

Silicon dioxide nano-powder (10-20 nm particle), chitosan (deacetylated chitin) and sodium tri-polyphosphate (TPP) were procured from Sigma-Aldrich Co., USA. Chitosan (0.4%, w/v) was dissolved in acetic acid (0.5%) prepared with HPLC-quality water on a magnetic stirrer with continuous stirring at 100 rpm. Then, the solution was filtered using Whatman filter papers (125 mm). TPP (0.2%) was also dissolved in HPLC-quality water and filtered. TPP solution was dropped slowly in the chitosan solution with continuous stirring at 100 rpm. The resulting chitosan nano-particles (NC) as a suspension were purified through centrifugation at 10000 g for 10 min. Then, NC particles were washed with HPLC-quality water three times. The supernatant was discarded and NC was stored for further use. Similarly, NC-NS was prepared by ionic gelation method where the concentration of chitosan (0.4%, w/v) TPP (0.2%, w/v), and Si (0.1% w/v) were used in the present experiment. NC (0.4%, w/v) was prepared along with NS (0.1% w/v) under magnetic stirrer (100 rpm) to obtain NC-NS particles. In brief, the NS (0.1% w/v) solution was prepared using HPLC-quality water and filtered. The NS solution was dropped slowly in the chitosan solution (0.4%, w/v) along with TPP (0.2%, w/v) under magnetic stirrer (100 rpm). NC-NS particles were collected through centrifugation and washed using HPLC-quality water for further experiment [22]. The TEM images of NC-NS particles are shown in Fig. 6.

## Plant cultivation and treatment

This study was designed with four treatments, including distilled water (control), NC, NS, and NC-NS foliar application. With the purpose of determination of the best treatment to alleviate the adverse effect of salinity stress, the foliar-sprayed plants were irrigated with NaCl at 0 (distilled water) and 100 mM levels. The seeds of wheat (*Triticum aestivum* L.) cultivar of Chamran was obtained from Agricultural Jahad Behbahan institute, Khuzestan, Iran. Among the wheat cultivars, Chamran is one of the most widely available variety and is cultivated in various regions of Iran. The seeds of similar size were sterilized in ethanol (70 % v/v) for 1 min, and in sodium hypochlorite (20% v/v) for 20 min, and then were washed with sterile deionized water three times. Ten seeds were sown in every pot (2 L) filled with equal amounts of perlite and soil. After emerging two leaves, they were reduced to three uniform plants per pot. The experiment was designed as a completely randomized block in a split-plot with the treatment as the main plot and NaCl irrigation as subplot. Each main plot consisted of eight plots and there were four replications per NaCl irrigation. The 15 days plants were foliar sprayed with 0 (distilled water), NC (0.05% w/v), NS (0.05% w/v) and NC-NS (0.1% w/v) solution. The solutions and distilled water were applied uniformly to the leaves of wheat plants as a fine spray using an atomizer. The foliar spray was carried out weekly for 28 days. Two days after the first treatment, the plants were irrigated with 0 (distilled water) and 100 mM of NaCl solution every 5 days. Salinity irrigation continued for one month. The control plants were irrigated with distilled water throughout the experiment. The plants were harvested three days after the last day of NaCl irrigation (50 days after sowing).

## Physiological and biochemical analysis

Fresh leaves and roots were used to measure H_2_O_2_ by spectrophotometer [44]. The malondialdehyde (MDA) content of fresh leaves and roots was determined based on the thiobarbituric acid assay [45]. The protein of fresh leaves and roots was extracted using sodium phosphate buffer and measured based on the Bradford [46] method. The activity of enzymes were assessed according to the standard procedures developed for catalase (CAT) (EC 1.11.1.6) [47], and ascorbate peroxidase (APX) (EC 1.11.1.11) [48], superoxide dismutase (SOD) (EC 1.15.1.1) [49], phenylalanine ammonia-lyase (PAL) (EC 4.3.1.24) [50], polyphenol oxidase (PPO) (EC 1.10.3.1) [33], and guaiacol peroxidase (POD) (EC 1.11.1.7) [51].

The total antioxidant power (FRAP) was measured in the fresh leaves and roots according to Szôllôsi et al [52] assay. The phenol content of fresh leaves and roots was measured using Folin's reagent [53]. The total flavonoids of fresh leaves and roots were colorimetrically measured as already described by Zhishen et al [54]. The anthocyanins of fresh leaves and roots were measured based on Wagner [55] assay. The fresh leaves and roots were with sulfosalicylic acid and an acidic ninhydrin solution was used to determine proline content [56]. The free amino acid contents in the fresh leaves and roots were determined based on the Yemm et al [57]. method. α-Tocopherol was extracted from the fresh leaves and roots and assayed as described by Baker et al [58]. The photosynthetic pigments of fresh leaves were extracted and measured based on the Wellburn [59] assay. The carbohydrate of dry leaves and roots were extracted and determined based on the phenol-sulfuric-acid procedure [60]. The glucose content in the dry leaves and roots was determined using a glucose assay kit (Sigma). The glycine betaine content in the fresh leaves and roots was assayed based on the Grieve et al. [61] method.

## Statistical analyses

The plant cultivation and treatment were done with four pots containing three plants per treatment. A set of three plants was considered as one replicate for each treatment. The values presented are the means of three independent replicates. The data were analyzed using SPSS statistical (version 24) package. Treatment means were subjected to factorial ANOVA and significant differences from control data were measured by Tukey’s test at *p*<0.05 significance level. Error bars represent standard deviation, and significant differences are shown by superscripted letters above each column in the figures.

## Data Availability

The datasets used and/or analyzed during the current study are available from the corresponding author upon reasonable request.

## Abbreviations

APX: Ascorbate peroxidase
CAT: Catalase
Chl: Chlorophyll
FAA: Free amino acids
GB: Glycine betaine
MDA: Malondialdehyde
NC: Nano-chitosan
NS: Nano-silicon
SOD: Superoxide dismutase
PAL: Phenylalanine ammonia-lyase
POD: Guaiacol peroxidase
PPO: Polyphenol oxidase
TPP: Tripolyphosphate
WSC: Water-soluble carbohydrates
